# Efficacy of Combination of Antiviral Therapy With Neutralizing Monoclonal Antibodies for Recurrent Persistent SARS-CoV-2 Pneumonia in Patients With Lymphoma

**DOI:** 10.1155/2024/8182887

**Published:** 2024-08-06

**Authors:** Xiaoyan Gai, Xiaoyan Sun, Beibei Liu, Wei Yan, Zikang Sheng, Qingtao Zhou, Yongchang Sun

**Affiliations:** Department of Pulmonary and Critical Care Medicine Peking University Third Hospital, Beijing 100083, China

**Keywords:** CD20 monoclonal antibody, immunocompromised patient, lymphoma, neutralizing antibodies, SARS-CoV-2 pneumonia

## Abstract

Despite the potential of neutralizing antibodies in the management of severe acute respiratory syndrome coronavirus-2 (SARS-CoV-2), clinical research on its efficacy in Chinese patients remains limited. This study is aimed at investigating the therapeutic effect of combination of antiviral therapy with neutralizing monoclonal antibodies for recurrent persistent SARS-CoV-2 pneumonia in patients with lymphoma complicated by B cell depletion. A prospective study was conducted on Chinese patients who were treated with antiviral nirmatrelvir/ritonavir therapy and the neutralizing antibody tixagevimab–cilgavimab (tix-cil). The primary outcome was the rate of recurrent SARS-CoV-2 infection. Five patients with lymphoma experienced recurrent SARS-CoV-2 pneumonia and received tix-cil treatment. All patients had a history of CD20 monoclonal antibody use within the year preceding SARS-CoV-2 infection, and two patients also had a history of Bruton's tyrosine kinase (BTK) inhibitor use. These patients had notably low lymphocyte counts and exhibited near depletion of B cells. All five patients tested negative for serum SARS-CoV-2 IgG and IgM antibodies. None of the patients developed reinfection with SARS-CoV-2 pneumonia after antiviral and tix-cil treatment during the 6-month follow-up period. In conclusion, the administration of antiviral and SARS-CoV-2-neutralizing antibodies showed encouraging therapeutic efficacy against SARS-CoV-2 pneumonia in patients with lymphoma complicated by B cell depletion, along with the potential preventive effect of neutralizing antibodies for up to 6 months.

## 1. Introduction

Patients with hematologic malignancies, such as lymphoma, are particularly vulnerable to SARS-CoV-2 caused by severe acute respiratory syndrome coronavirus-2 (SARS-CoV-2). They are at a heightened risk of severe illness, mortality, and recurrent SARS-CoV-2 pneumonia [1, 2]. This population also experiences prolonged viral shedding, with possible negative consequences for withdrawing or postponing chemotherapy [3, 4]. After undergoing chemotherapy, these patients exhibit suppressed cellular and humoral immune responses, posing a challenge in generating SARS-CoV-2 antibodies, even after vaccination. Approximately half of patients with lymphoma struggle to produce effective SARS-CoV-2 antibodies [5], particularly those undergoing B cell depletion therapies such as anti-CD20 monoclonal antibodies and Bruton's tyrosine kinase (BTK) inhibitor. The inhibitory effects of B cell depletion therapy can persist for up to 12 months, presenting significant obstacles to the treatment of SARS-CoV-2 [4, 5].

SARS-CoV-2, a spherical RNA virus, binds to the human angiotensin-converting enzyme-2 (ACE-2) receptor via its spike glycoprotein, initiating infection. Moreover, the spike glycoprotein also interacts with the toll-like receptor 4 (TLR4) leading to the induction of strong proinflammatory responses in the lungs [6, 7]. Despite recent advancements in various preventive and therapeutic strategies, including SARS-CoV-2 vaccination, oral antiviral drugs, and convalescent plasma therapy, some patients with lymphoma still experience difficulty in achieving sustained immunity. They struggle to eliminate the virus from their bodies and may suffer from recurrent episodes of SARS-CoV-2 pneumonia. Neutralizing monoclonal antibodies targeting the receptor-binding domain (RBD) of the SARS-CoV-2 spike protein have been isolated from recovered individuals and have shown promising results in preventing and treating SARS-CoV-2 infection [1, 2]. Moreover, modifications to optimize the Fc segment of antibodies have extended their half-life, resulting in sustained antibody levels for 6–12-month postadministration [1, 2]. This provides a potential therapeutic strategy for treating SARS-CoV-2 pneumonia in these patients and preventing reinfection.

In countries such as the United States and France, the SARS-CoV-2-neutralizing antibody tixagevimab–cilgavimab (tix-cil; Evusheld™, AstraZeneca, Sweden) has obtained emergency use authorization (EUA) for individuals aged 12 years and older with moderate-to-severe immunodeficiency who may not mount an adequate vaccine response [2]. However, the utilization of SARS-CoV-2-neutralizing antibodies is presently limited to the Boao region of Hainan Province, China, where patients must self-fund their treatment. Presently, there is limited research available on the efficacy of this approach.

This study sought to present the characteristics of five lymphoma patients with recurrent SARS-CoV-2 pneumonia who were treated with combination of antiviral therapy with neutralizing monoclonal antibodies and to provide a comprehensive overview and analysis of treatment outcomes and follow-up observations in these patients following the administration of combined therapy. Based on the literature and our multidisciplinary experience, the combination multitarget therapy, such as antiviral therapy coupled with monoclonal antibodies, showed encouraging preventive and therapeutic efficacy against SARS-CoV-2 pneumonia in this specific patient population.

## 2. Materials and Methods

### 2.1. Ethical Clearance

The study was conducted in accordance with the principles of the Declaration of Helsinki and received approval from the Ethics Committee of Peking University Third Hospital (approval number: 2023-006-01). Informed consent was obtained from all participants.

### 2.2. Study Design and Patient Enrollment

This was a prospective study involving Chinese patients admitted to the Department of Respiratory and Critical Care Medicine at Peking University Third Hospital from February 1, 2023, to April 30, 2023. Inclusion criteria are as follows: (1) comprised patients with lymphoma and recurrent SARS-CoV-2 pneumonia, defined as experiencing two or more episodes within the past 6 months, with radiographic clearing between episodes; (2) participants who received antiviral therapy but continued to experience recurrent COVID-19 pneumonia; (3) individuals with undetectable or near-zero levels of SARS-CoV-2 IgG-IgM antibodies in the serum; and (4) patients willing to undergo combination of antiviral and SARS-CoV-2-neutralizing antibody treatment. Exclusion criteria included severe allergic conditions or contraindications to COVID-19 antibody therapy, as well as patients unwilling to receive combined therapy.

### 2.3. Diagnosis of SARS-CoV-2 Pneumonia

SARS-CoV-2 pneumonia diagnosis followed established guidelines [8, 9]. Confirmation of SARS-CoV-2 infection relied on reverse-transcriptase-polymerase-chain-reaction (RT-PCR) assay (Biogerm Biotechnology Co., Ltd., Shanghai, China) on throat swabs and/or bronchoalveolar lavage fluid (BALF), supplemented by characteristic imaging features observed on chest computed tomography (CT) scans.

### 2.4. Administration of Combined Therapy of Antiviral and Neutralizing Antibodies

Eligible patients received combined treatment of oral antiviral nirmatrelvir/ritonavir (Paxlovid, Pfizer) and tix-cil (Evusheld, AstraZeneca, Sweden) at a dosage of 600 mg via intramuscular injection.

### 2.5. Data Collection

Comprehensive patient information, including demographics, lymphoma history, and SARS-CoV-2 pneumonia details, was collected. Additional data encompassed chest imaging results, routine blood tests, lymphocyte subsets, specifics of SARS-CoV-2 pneumonia treatment, and serum IgG-IgM antibody level postneutralizing antibody administration. Antibody testing utilized an IgG/IgM Antibody Detection Kit for SARS-CoV-2, employing magnetic particle chemiluminescence (manufactured by Anto Biotechnology Co., Ltd., Zhengzhou, China), with positivity defined as S/CO ≥ 1.0. Follow-up observations monitored patient conditions, reinfection occurrences, and subsequent serum SARS-CoV-2 antibody levels. Quantitative variables are expressed as median (minimum–maximum).

## 3. Results

### 3.1. Patient Characteristics

Six patients with lymphoma who had recurrent SARS-CoV-2 pneumonia from February 1, 2023, to April 30, 2023, met the study's inclusion criteria. Among these patients, five were enrolled in this study and received combined therapy of antiviral and tix-cil treatment; one patient declined antibody therapy and was subsequently excluded from the study.

Among the included patients, three were male, whereas two were female, averaging 51.8 years (range: 36–59 years). All five lymphomas were non-Hodgkin lymphoma. The lymphoma subtypes consisted of three cases of follicular lymphoma, one of mucosa-associated lymphoid tissue lymphoma, and one of mantle cell lymphoma. All patients had previously received anti-CD20 monoclonal antibody treatment for lymphoma, and the time interval between the last treatment and the onset of SARS-CoV-2 pneumonia was less than 1 year, averaging 2.3 months (range: 0.5–4 months). Two patients also underwent BTK treatment, with time intervals ranging from 0.5 to 4 months between the initial positive SARS-CoV-2 test and the last BTK treatment (Table 1). Upon admission, routine blood tests revealed decreased lymphocyte levels, with an absolute lymphocyte count of 0.53 × 10^9^/L (range 0.37–0.89 × 10^9^/L; reference range: 1.1–3.2 × 10^9^/L). Lymphocyte subset analysis showed that B cells were nearly depleted, averaging 0.22 cells/*μ*L (range: 0–0.43 cells/*μ*L; reference range: 80–616 cells/*μ*L) (Table 1).

### 3.2. SARS-CoV-2 Pneumonia Presentation and Treatment

Among the five patients, only one received two doses of the SARS-CoV-2 vaccine, while the remaining four were unvaccinated. All patients experienced their initial SARS-COV-2 infection between December 2022 and January 2023, a period characterized by China's transition from a strict zero-tolerance policy to a surge in infections. The most frequently observed symptoms at presentation were fever (5/5), fatigue (5/5), cough (4/5), and dyspnea (1/5). Chest CT scans of all patients revealed bilateral ground-glass opacities, and their throat swabs tested positive for SARS-CoV-2 viral nucleic acid, meeting the diagnostic criteria for moderate SARS-CoV-2 pneumonia. All five patients experienced recurrent episodes of SARS-CoV-2 pneumonia, with two patients having a second episode and three patients experiencing a third episode. Clinical symptoms included recurrent fever without respiratory distress or mild respiratory difficulty. Four patients underwent BALF pathogen metagenomics next-generation sequencing (NGS) (mNGS) via bronchoscopy [10], confirming SARS-CoV-2 infection. The viral subtypes identified were BF.7.14 in two cases and BA.5.2.48 in one case, and the subtype of the remaining patient could not be determined due to limited sequencing data. Upon admission, all patients tested negative for SARS-CoV-2 IgG and IgM antibodies. Furthermore, the five patients have consistently tested negative for serum SARS-CoV-2 IgG and IgM antibodies since their initial infection with SARS-CoV-2.

All patients received oral antiviral nirmatrelvir/ritonavir (Paxlovid, Pfizer) treatment and neutralizing antibody therapy during hospitalization. Four patients received low-dose steroid treatment, and three underwent convalescent plasma infusion.

### 3.3. Imaging Characteristics

**Figure 1:**
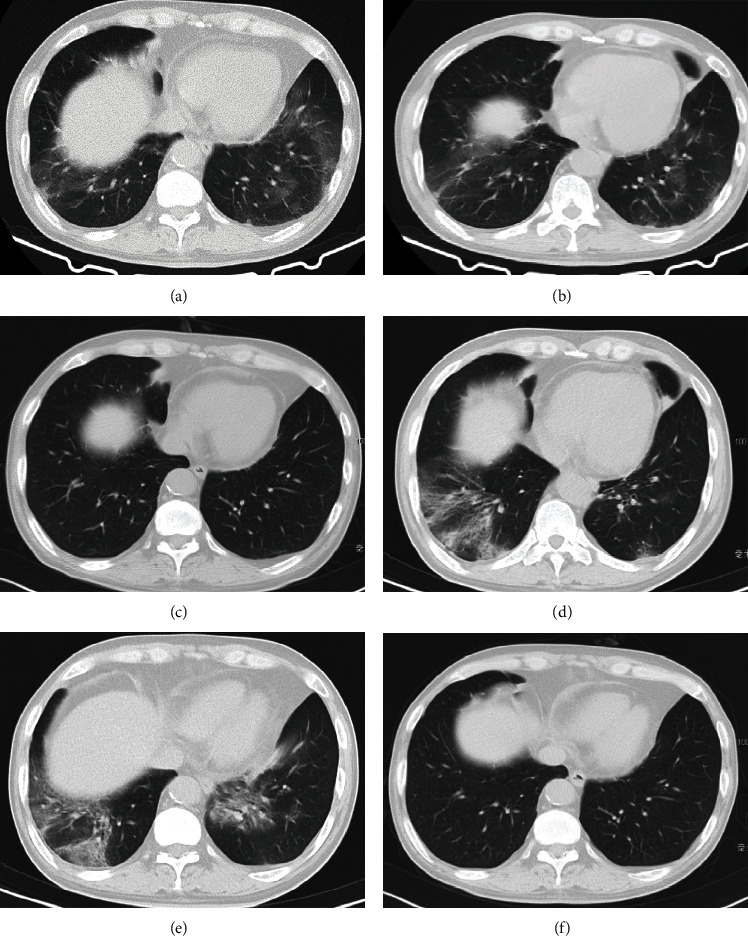
Chest CT scans of the patient with recurrent SARS-CoV-2 pneumonia. (a) First episode of SARS-CoV-2 pneumonia. (b) Second episode of SARS-CoV-2 pneumonia, and (c) improvement after treatment. (d, e) Third episode of SARS-CoV-2 pneumonia, and (f) improvement after treatment with Paxlovid and tix-cil.

The predominant lung imaging results showed bilateral ground-glass opacities, mainly located in subpleural regions. These opacities have unclear borders and are frequently distributed across multiple lung lobes. No consolidation, crazy-paving pattern, or pleural effusion was observed (Figure 1).

The patient (Case 1) is a 57-year-old male and experienced three episodes of SARS-CoV-2 pneumonia during the past 4 months.

On December 4, 2022, the patient developed fever, and a throat swab test showed a positive result for the SARS-CoV-2 antigen. Chest CT revealed bilateral ground-glass opacities in the lower lungs (Figure 1(a)). The patient was treated with oral antipyretic medication, and 13 days later, a follow-up throat swab test showed that the SARS-CoV-2 antigen turned negative.

On January 28, 2023, the patient experienced a second episode of fever for 10 days, and chest CT revealed newly developed inflammatory infiltrates in both lungs (Figure 1(b)). NGS analysis of the BALF confirmed the presence of SARS-CoV-2 (sequence count: 1524). The patient was treated with oral methylprednisolone and Paxlovid, and the body temperature returned to normal. Two weeks later, follow-up lung CT showed significant resolution of the pneumonia (Figure 1(c)).

On March 2, 2023, the patient experienced a third episode of fever. Chest CT revealed multiple new patchy areas of increased density in both lungs, indicating worsening compared to previous scans (Figures 1(d) and 1(e)). Throat swab testing showed SARS-CoV-2 PT-PCR positive, with an ORF CT value of 32.1 and an N CT value of 29.86. NGS analysis of BALF confirmed the presence of SARS-CoV-2 (BF.7.14) with a sequence count of 37,782. After receiving treatment with oral Paxlovid for 10 days and a 600 mg injection of tix-cil and corticosteroids, the patient's body temperature normalized. Fatigue and decreased appetite gradually improved. Inflammatory markers, including C-reactive protein (CRP) levels, showed improvement. Additionally, follow-up lung CT scans 2 weeks later revealed complete resolution of lung inflammation (Figure 1(f)). The throat swab test for SARS-CoV-2 nucleic acid was negative.

At follow-up, the patient did not have SARS-CoV-2 pneumonia during the 6-month follow-up after tix-cil treatment.

### 3.4. Safety

No adverse events occurred after the intramuscular administration of a single dose of tix-cil.

### 3.5. Follow-Up

**Figure 2:**
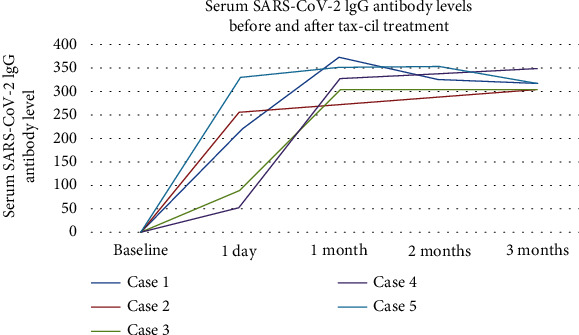
Serum SARS-CoV-2 IgG antibody levels before and after tax-cil treatment.

All five patients underwent 6-month follow-up. All patients exhibited successful development of SARS-CoV-2 antibodies following administration of a SARS-CoV-2-neutralizing antibody injection. The average level of SARS-CoV-2 IgG antibodies was 189 (range: 54–332) on 1 day after vaccination, 341 (range: 305–374) at 1 month, and 320 (range: 306–350) at 3 months (Table S1 and Figure 2). No patient experienced recurrent fever during the follow-up period. One patient reported a day of low-grade fever and sore throat 2 months after vaccination; this patient tested positive for SARS-CoV-2 antigen, which turned negative 5 days later. The lung CT scan showed normal results. No additional cases of SARS-CoV-2 pneumonia were observed in the remaining patients during the follow-up.

## 4. Discussion

In this study, we present the cases of five patients with lymphoma who experienced recurrent persistent SARS-CoV-2 pneumonia due to compromised immune function following chemotherapy. These patients exhibited several common features: (1) decreased lymphocyte levels in the blood, with a notable reduction in B cells; (2) negative IgG and IgM antibodies against the novel coronavirus in their serum; and (3) a history of CD20 monoclonal antibody therapy within the past year.

One significant predisposing factor for poor prognosis in patients with lymphoma with SARS-CoV-2 is the markedly low lymphocyte count [11]. A second shared feature is the antibody profile. Patients with recurrent SARS-CoV-2 infection consistently exhibit negative SARS-CoV-2 antibodies, which can be linked to underlying lymphoma and B cell therapies [12, 13]. Their humoral response is impaired, as indicated by reduced antibodies against the SARS-CoV-2 spike protein compared to healthy individuals [4]. This finding is particularly noteworthy given the correlation between the production kinetics of neutralizing antibodies and the clinical outcomes of the disease. A recent study showed that individuals who died from SARS-CoV-2 were delayed in releasing neutralizing antibodies compared to discharged patients with high levels of neutralizing antibodies [14]. Furthermore, the delayed release of neutralizing antibodies within the first 14 days after onset of SARS-CoV-2 and the delayed viral clearance were found to be correlated with fatal cases of SARS-CoV-2 [14].

The third commonality relates to anti-CD20 therapy. These findings align with existing literature indicating that patients with low B cell counts cannot effectively mount an antibody response against SARS-CoV-2 [15]. To combat recurrent infections and enhance resistance against the virus, we administered SARS-CoV-2-neutralizing antibody therapy. During a follow-up period of approximately 6 months, none of the patients experienced further episodes of SARS-CoV-2 pneumonia and maintained prolonged antibody levels. This suggests a beneficial protective effect of SARS-CoV-2-neutralizing antibodies in this immunocompromised population.

Lymphoma patients with compromised immune function are recognized as a high-risk group for infections [16], and their ability to clear the SARS-CoV-2 virus and their overall prognosis is often poor. In lymphomas originating from B cells, the humoral immune response is severely impaired, and targeted therapies directed at B cells, such as CD20 antibodies, BTK inhibitors, and CAR-T cells, may cause B cell depletion and further suppress humoral immunity. Consequently, the patients struggle to generate sufficient antibody responses even after vaccination [17–19]. CD20 monoclonal antibodies have been reported to serve as a fundamental treatment approach for many B cell malignancies; however, owing to the depletion of B cells, patients have a limited ability to generate SARS-CoV-2 antibodies, leading to challenges in viral clearance that may persist for weeks or result in recurrent SARS-CoV-2 infection [17, 18]. Chronic or recurrent SARS-CoV-2 infection can contribute to the emergence of viral variants, particularly in the spike protein of SARS-CoV-2, which enhances the virus's ability to evade the immune response. Moreover, the persistent positive SARS-CoV-2 RT-PCR is associated with an increased SARS-COV-2 mortality rate in lymphoma patients [20]. The management of SARS-CoV-2 pneumonia includes control of viral replication and facilitating the clearance of the virus.

In addition to direct-active antivirals, such as nirmatrelvir/ritonavir and molnupiravir, passive immunotherapy (monoclonal neutralizing antibody therapy and convalescent plasma therapy) is also an effective treatment approach for immunocompromised patients. SARS-CoV-2-neutralizing antibodies have demonstrated promising preventive and therapeutic effects in immunocompromised patients with SARS-CoV-2 pneumonia. Tix-cil, a combination of monoclonal antibodies, effectively inhibits viral entry into cells by targeting two distinct epitopes on the spike protein. The modification of the Fc region enhances antibody optimization, prolongs their half-life, and ensures sustained protection for weeks to months [21]. The PROVENT study revealed the prophylactic effect of tax-cil within a 6-month follow-up period [22]. Additionally, the TACKLE study showed that treating adult outpatient SARS-CoV-2 patients with tix-cil significantly decreased the risk of severe SARS-CoV-2 and death [23]. Stuver et al. [24] conducted a specific investigation of 52 patients with hematopoietic malignancies, most of whom had non-Hodgkin lymphoma (38.5%). Almost half of the participants (46.2%) had undergone hematopoietic stem cell transplantation or chimeric antigen receptor T-cell therapy. The study showed that tix-cil was an effective strategy for preventing SARS-CoV-2 infection and exhibited varying neutralizing capabilities against different Omicron subtypes. Recent literature supporting the use of combination therapy in B cell–depleted immunocompromised patients with persistent or relapsed SARS-CoV-2 pneumonia [25–26]. A prospective cohort study evaluated the efficacy and safety of antiviral plus antispike monoclonal antibody combination therapy versus monotherapy for high-risk immunocompromised patients with mild-to-moderate SARS-CoV-2 infection during the Omicron era. The study demonstrated that combination therapy with monoclonal antibody plus an antiviral was associated with a lower risk of COVID-19 progression compared to monotherapy [25]. Additionally, two other studies highlighted the importance of combination therapy, involving two antivirals (remdesivir and nirmatrelvir/ritonavir) alongside monoclonal antibodies, in effectively managing immunocompromised patients with prolonged or relapsed COVID-19 [26, 27]. These findings emphasize the need for adopting combination multitarget therapy, such as antiviral therapy coupled with monoclonal antibodies, to effectively mitigate persistent viral shedding and severe SARS-CoV-2 infection in immunocompromised individuals.

It is important to note that, despite observing effective results, compared to early SARS-CoV-2 strains, the spike protein of the Omicron variant has undergone continuous mutations, which may diminish the potency of antibodies developed against earlier strains. In the Davis et al. [28] study, even within 3–6 months of tix-cil treatment, few patients experienced breakthrough SARS-CoV-2 infection, primarily linked to viral variants and the emergence of new subtypes. Recent studies about the mAbs for treating SARS-CoV-2-infected patients depicted that combined therapy is more effective than monotherapy, and chimeric antibody maybe could provide hope for treating the Omicron variants [29, 30]. Given the highly mutable nature of the SARS-CoV-2 pathogen, future investigations should prioritize the development of broad-spectrum neutralizing antibody therapies targeting highly conserved regions of the spike protein.

The study has several limitations. First, the sample size is small, with only five cases. This limitation arises from the constrained accessibility of tix-cil within China, which hinders the enrollment of a larger patient cohort. Second, this is a single-center study, potentially impacting its generalizability. Third, the Omicron virus variant is undergoing continuous mutations, with the newly emerged XBB variant gaining prevalence. Further research is required to explore the preventative and therapeutic efficacy of tix-cil antibodies against the XBB variant.

## 5. Conclusions

Patients with lymphoma, especially those treated with anti-CD20 monoclonal antibodies that result in B cell depletion, experience difficulty in clearing the virus after SARS-CoV-2, leading to recurrent persistent infections. The combination of antiviral therapy with tix-cil, a SARS-CoV-2-neutralizing antibody, provides effective therapeutic benefits for immunocompromised patients with SARS-CoV-2 pneumonia. In the future, further studies should investigate the effect of neutralizing antibodies on the treatment of new mutant strains of SARS-CoV-2 and explore the development of neutralizing antibodies targeting highly conserved regions of the spike protein if necessary.

## Figures and Tables

**Table 1:** Baseline characteristics of patients with recurrent SARS-CoV-2 pneumonia and lymphoma who underwent tix-cil treatment.

**No.**	**Sex**	**Age**	**Symptom**	**Duration of recurrent SARS-CoV-2**	**Lymphoma subtypes**	**Anti-CD20 therapy**	**BTK inhibitor therapy**	**Time interval between initial SARS-CoV-2 pneumonia and the last anti-CD20 treatment**	**Time interval between initial SARS-CoV-2 pneumonia and the last BTK treatment**	**WBC in CBC (×10** ^**9**^ **/L)**	**Absolute lymphocyte count in CBC (×10** ^**9**^ **/L)**	**Absolute B cell count in the lymphocyte subpopulation of peripheral blood (/*μ*L)**	**Percentage of B cells in the lymphocyte subpopulation of peripheral blood**	**Serum anti-SARS-CoV-2 antibodies**
Case 1	Male	57	Intermittent fever, cough	3 months	MALT	Yes	No	2 months	N/A	4.57	0.34	0.27	0.04	Negative
Case 2	Male	36	Intermittent fever, cough	3 months	Follicular lymphoma	Yes	No	3 months	N/A	4.59	0.7	0	0	Negative
Case 3	Female	59	Intermittent fever, cough	4 months	Mantle cell lymphoma	Yes	Yes	4 months	4 months	3.42	0.94	0.43	0.04	Negative
Case 4	Female	48	Intermittent fever, cough, dyspnea	3 months	Follicular lymphoma	Yes	No	2 months	N/A	5.53	0.29	0	0	Negative
Case 5	Male	59	Intermittent fever, cough	3 weeks	Follicular lymphoma	Yes	Yes	2 weeks	2 weeks	2.2	0.37	0.41	0.04	Negative

Abbreviations: BTK, Bruton's tyrosine kinase; CBC, complete blood count; NA, not available; SARS-CoV-2, severe acute respiratory syndrome coronavirus-2; tix-cil, tixagevimab–cilgavimab; WBC, white blood cell.

## Data Availability

The original contributions presented in the study are included in the article. Further inquiries can be directed to the corresponding author.

## Supporting Information

Supporting Information Additional supporting information can be found online in the Supporting Information section. Table S1. SARS-CoV-2 IgG antibody levels in serum before and after tax-cil treatment.
