# Supplementary material for: Efficacy of Combination of Antiviral Therapy With Neutralizing Monoclonal Antibodies for Recurrent Persistent SARS-CoV-2 Pneumonia in Patients With Lymphoma
Source: Biomed Res Int. 2024 Aug 6;2024:8182887. doi: 10.1155/2024/8182887 (PMC11321881; doi:10.1155/2024/8182887)
Supplement: Supporting Information — Additional supporting information can be found online in the Supporting Information section. Table S1. SARS-CoV-2 IgG antibody levels in serum before and after tax-cil treatment. [file 8182887.f1.pdf]

Supplementray Table 1. SARS-CoV-2 IgG Antibody Levels in Serum Before and After Tix-cil Treatment

No	Baseline	1 day	1 month	2 months	3 months
case 1	0	217	374	328	320
case 2	0	257	NA	NA	306
case 3	0	87	305	NA	306
case 4	0	54	330	NA	350
case 5	0	332	355	355	322
